# Changes in EEG Power Spectral Density and Cortical Connectivity in Healthy and Tetraplegic Patients during a Motor Imagery Task

**DOI:** 10.1155/2009/279515

**Published:** 2009-06-24

**Authors:** Filippo Cona, Melissa Zavaglia, Laura Astolfi, Fabio Babiloni, Mauro Ursino

**Affiliations:** ^1^Department of Electronics, Computer Science and Systems, University of Bologna, Via Venezia 52, 47023 Cesena, Italy; ^2^Department of Human Physiology and Pharmacology, Sapienza University of Rome, 00185 Rome, Italy; ^3^Istituti di ricovero e cura a carattere scientifico (IRCCS) Fondazione Santa Lucia, 00179 Rome, Italy

## Abstract

Knowledge of brain connectivity is an important aspect of modern neuroscience, to understand how the brain realizes its functions. In this work, neural mass models including four groups of excitatory and inhibitory neurons are used to estimate the connectivity among three cortical regions of interests (ROIs) during a foot-movement task. Real data were obtained via high-resolution scalp EEGs on two populations: healthy volunteers and tetraplegic patients. A 3-shell Boundary Element Model of the head was used to estimate the cortical current density and to derive cortical EEGs in the three ROIs. The model assumes that each ROI can generate an intrinsic rhythm in the beta range, and receives rhythms in the alpha and gamma ranges from other two regions. Connectivity strengths among the ROIs were estimated by means of an original genetic algorithm that tries to minimize several cost functions of the difference between real and model power spectral densities. Results show that the stronger connections are those from the cingulate cortex to the primary and supplementary motor areas, thus emphasizing the pivotal role played by the CMA_L during the task. Tetraplegic patients exhibit higher connectivity strength on average, with significant statistical differences in some connections. The results are commented and virtues and limitations of the proposed method discussed.

## 1. Introduction

It is well known that the execution of even simple motor and/or cognitive tasks by the brain requires the participation of multiple cortical regions, which are mutually interconnected and exchange their information via plastic long-range synapses. Consequently, knowledge of brain connectivity is becoming an essential aspect of modern neuroscience, especially useful to understand how the brain realizes its basic functions and what the role of the different regions is. Connectivity, however, is an elusive concept, which can have different definitions depending on the emphasis of the investigators [1]. In particular, the definition of connectivity is strictly related to the mathematical method used to extract connectivity parameters from data, that is, it is “model dependent” and should always be used together with the particular method adopted. For instance, most methods presently used to derive connectivity graphs (such as the Direct Transfer Function or the Partial Directed Coherence [2–8]) are based on the assumption of linearity, whereas neurons are intrinsically nonlinear. Moreover, these methods use empirical equations (i.e., they are based on black box models), which do not provide a description of the underpinning physiological mechanisms (e.g., they do not explicitly consider the time constant and strength of synapses, the role of inhibitory interneurons, etc.). On the other hand, the main advantage of these methods is that they provide analytical solutions to the problem, which are not “modeler driven.”

As an alternative method to study effective connectivity, a few authors in recent years have employed the so-called “neural mass models.” These models were originally proposed in the mid seventies [9, 10] and subsequently improved in the late nineties [11, 12]. They mimic the activity of entire neural populations via the feedback arrangement of excitatory and inhibitory groups, which are assumed to share a similar membrane potential and work in synchronism. The interaction between excitatory and inhibitory groups can produce oscillatory rhythms, either via an intrinsic instability of the model (like a limit cycle) or by a resonance amplification of an external noise. In particular, similar models have been used to simulate alpha rhythms [11], dynamics in the olphactory cortex [13], or paradoxical epileptic discharges [12, 14]. A few recent studies used these models to study effective connectivity among different regions of interest (ROIs), to analyze the dependence of cortical EEG on connectivity patterns [15, 16] and to evaluate the EEG power spectral density [17]. Recently, we also used neural mass models, including fast inhibitory dynamics, to simulate the power spectral density of cortical EEG [18–20] during simple motor tasks. The main indication of these studies is that neural populations with different dynamics (e.g., different time constants of excitatory and inhibitory synapses) suitably interconnected, can produce EEG rhythms similar to those measured in human subjects via high-resolution EEG methods.

Application of neural mass models to estimate effective connectivity is, however, a very hard task, due to the elevated number of parameters involved and the presence of nonlinear terms, which preclude the use of analytical solutions. For instance, in a recent paper [21] we derived some connectivity patterns between three cortical regions (the cyngulate and the primary and supplementary motor cortices) during a simple foot-movement task, by minimizing a least-square criterion function of the difference between model and data spectral densities. However, just a few exemplary cases could be analyzed, since the minimization algorithm often converges to a suboptimal solution (i.e., a local minimum) which may exhibit just a poor fitting and, moreover, may be characterized by unphysiological parameter values. Furthermore, also the metrics used to compare model and patient spectral densities may be questionable and affect the final minimisation results.

For this reason, in the present paper we designed a new method, based on a genetic algorithm, to provide an automatic fitting between model and real data. The method tries to find absolute minima of alternative cost functions within the same procedure. Genetic algorithms have already been used to estimate the parameters of a neural mass model in order to fit real data (see, e.g., [22]). The algorithm has been applied to high-resolution scalp EEG data measured during a simple foot-movement task; scalp EEG was preliminarily propagated to the cortex via a propagation model, to infer cortical electrical activity in three Regions of Interest (ROIs). The model [20] assumes that each ROI is characterized by an intrinsic rhythm (established by the time constants of synapses) and can receive additional rhythms from other connected ROIs. Results have been applied to a group of normal subjects and a group of tetraplegic patients to establish simple patterns of connectivity between the cyngulate, motor, and premotor cortices, and to look for possible differences in the two populations.

## 2. Method

### 2.1. Model of a Single Population

The model of a single population was obtained by modifying equations proposed by Wendling et al. [12]. It consists of four neural groups which communicate via excitatory and inhibitory synapses: pyramidal cells, excitatory interneurons, inhibitory interneurons with slow synaptic kinetics, and inhibitory interneurons with faster synaptic kinetics. Each neural group simulates a pool of neurons which are lumped together and which are assumed to receive similar input and to behave in a similar manner. One lumped circuit communicates with another through the average firing rate corresponding to what that given population of cells is firing on average.

Each neural group receives an average postsynaptic membrane potential from the other groups, and converts the average membrane potential into an average density of spikes fired by the neurons. This conversion is simulated via a static sigmoidal relationship. The effect of the synapses is described via second-order linear transfer functions, which convert the presynaptic spike density into the postsynaptic membrane potential. Three different kinds of synapses, with impulse response *h*_*e*_, *h*_*i*_, and *h*_*g*_, are used to describe the synaptic effect of excitatory neurons (both pyramidal cells and excitatory interneurons), of slow inhibitory interneurons and of fast inhibitory interneurons, respectively. Model equations can be written as follows.

**Pyramidal Neurons:**

(1) $$\frac{dy_{0}\left(t\right)}{dt}=y_{5}\left(t\right),$$

(2) $$\frac{dy_{5}\left(t\right)}{dt}=A\cdot a_{1}\cdot z_{0}\left(t\right)-2\cdot a_{1}\cdot y_{5}\left(t\right)-a_{1}^{2}\cdot y_{0}\left(t\right),$$

(3) $$z_{0}\left(t\right)=\frac{\left(2\cdot e_{0}\right)}{1+e^{r\cdot \left(s_{0}-v_{0}\right)}},$$

(4) $$v_{0}\left(t\right)=C_{2}\cdot y_{1}\left(t\right)-C_{4}\cdot y_{2}\left(t\right)-C_{7}\cdot y_{3}\left(t\right).$$

**Excitatory Interneurons:**

(5) $$\begin{array}{c} \frac{dy_{1}\left(t\right)}{dt}=y_{6}\left(t\right), \end{array}$$

(6) $$\begin{array}{c} \frac{dy_{6}\left(t\right)}{dt}=A\cdot a_{1}\cdot \left(z_{1}\left(t\right)+\frac{p\left(t\right)}{C_{2}}\right)-2\cdot a_{1}\cdot y_{6}\left(t\right)-a_{1}^{2}\cdot y_{1}\left(t\right), \end{array}$$

(7) $$\begin{array}{c} z_{1}\left(t\right)=\frac{\left(2\cdot e_{0}\right)}{1+e^{r\cdot \left(s_{0}-v_{1}\right)}}, \end{array}$$

(8) $$\begin{array}{c} v_{1}\left(t\right)=C_{1}\cdot y_{0}\left(t\right). \end{array}$$

**Slow Inhibitory Interneurons:**

(9) $$\begin{array}{c} \frac{dy_{2}\left(t\right)}{dt}=y_{7}\left(t\right), \end{array}$$

(10) $$\begin{array}{c} \frac{dy_{7}\left(t\right)}{dt}=B\cdot b_{1}\cdot z_{2}\left(t\right)-2\cdot b_{1}\cdot y_{7}\left(t\right)-b_{1}^{2}\cdot y_{2}\left(t\right), \end{array}$$

(11) $$\begin{array}{c} z_{2}\left(t\right)=\frac{\left(2\cdot e_{0}\right)}{1+e^{r\cdot \left(s_{0}-v_{2}\right)}}, \end{array}$$

(12) $$\begin{array}{c} v_{2}\left(t\right)=C_{3}\cdot y_{0}\left(t\right). \end{array}$$

**Fast Inhibitory Interneurons:**

(13) $$\begin{array}{c} \frac{dy_{3}\left(t\right)}{dt}=y_{8}\left(t\right), \end{array}$$

(14) $$\begin{array}{c} \frac{dy_{8}\left(t\right)}{dt}=G\cdot g_{1}\cdot z_{3}\left(t\right)-2\cdot g_{1}\cdot y_{8}\left(t\right)-g_{1}^{2}\cdot y_{3}\left(t\right), \end{array}$$

(15) $$\begin{array}{c} z_{3}\left(t\right)=\frac{\left(2\cdot e_{0}\right)}{1+e^{r\cdot \left(s_{0}-v_{3}\right)}}, \end{array}$$

(16) $$\begin{array}{c} v_{3}\left(t\right)=C_{5}\cdot y_{0}\left(t\right)-C_{6}\cdot y_{2}\left(t\right). \end{array}$$

In these equations, the symbols *v*_*i*_ represent the average membrane potentials (*i* = 0, 1, 2, 3 for the four groups). These are the input for the sigmoid function which converts them into the average density of spikes (*z*_*i*_, *i* = 0, 1, 2, 3) fired by the neurons. Then, these outputs enter into the synapses (excitatory, slow inhibitory, or fast inhibitory), represented via the second-order linear functions. Each synapse is described by an average gain (*A*, *B*, *G* for the excitatory, slow inhibitory, and fast inhibitory synapses, resp.) and a time constant (the reciprocal of *a*_1_, *b*_1_, and *g*_1_, resp.). The outputs of these equations, which can be excitatory, slow inhibitory, or fast inhibitory, represent the postsynaptic membrane potentials (*y*_*i*_, *i* = 0, 1, 2, 3). Interactions among neurons are represented via seven connectivity constants (*C*_*i*_). Finally, *p*(*t*) represents all exogenous contributions, both excitation coming from external sources and the density of action potentials coming from other connected regions.

### 2.2. Model of Connectivity Among ROIs

The previous model was used to simulate a single ROI, the dynamic of which ensues from the interactions among the four neural subgroups. In order to study how the ROIs interact, we consider N ROIs which are interconnected through long-range excitatory connections. To simulate this connectivity we assumed that the average spike density of pyramidal neurons (*z*_0_) affects the input *p*(*t*) in (6) via a weight factor, *W*, and a time delay, *T*. Hence, the input *p*_*i*_(*t*) in the *i*th ROI can be computed as follows:

(17) $$p_{i}\left(t\right)=n_{i}\left(t\right)+\underset{j}{\sum }W_{ij}z_{0,j}\left(t-T\right),$$

where *W*_*ij*_ is the weight of the synaptic link from the *j*th (presynaptic) ROI to ith (postsynaptic) ROI, *T* is the time delay (assumed equal for all synapses), *n*_*i*_(*t*) represents a gaussian white noise with mean value *m*_*i*_ and standard deviation *σ*_*i*_, and the sum in the right hand member of (17) is extended to all ROIs, *j*, which target into the ROI *i*.

### 2.3. Acquisition and Processing of EEG Data

The experiment took place in the laboratories of the Santa Lucia Foundation, Rome, after the informed consent was obtained. The subject was comfortably seated in an armchair with both arms relaxed, in an electrically shielded, dimly lit room. He was asked to perform a brisk protrusion of the lips (lip pursing) while he was performing a right foot movement. A 58-channel EEG system (BrainAmp, Brainproducts GmbH, Germany) was used to record electrical potentials by means of an electrode cap, accordingly to an extension of the 10–20 international system. A/D sampling rate was 200 Hz. During motor task, subject was instructed to avoid eye blinks, swallowing, or any movement other than the required foot movements. Bipolar EMG was recorded from control and spinal cord injury (SCI) subjects, with surface electrodes from the right tibialis anterior muscle and orbicularis oris muscle to detect the onset of foot and lip movements, respectively. The electro-oculograms (EOGs) were recorded to avoid trials with artifacts due to eye-blink movements. The EMG was monitored throughout recordings from electrodes placed as described above to avoid poor quality of the recordings due to muscular artifacts. Artifact rejection was performed on a wide segmentation of the trials (from −4.0 s to +4.0 s) while a narrow segmentation (from −2.5 s to +0.5 s) was used as analysis period.

A 3-shell Boundary Element Model (BEM) of the head was used to estimate the cortical current density (CCD) distribution in some regions of interest (ROI) of the cortex (the cingulate cortex (CMA_L), the primary motor area (M1F_L), and the supplementary motor area (SMAp_L) starting from activity measured on the scalp. The procedure used is described in previous works [18, 19, 23]. From the CCD, the average estimated cortical activity in the region has then been evaluated. The latter has been successively subjected to spectral analysis in order to produce the spectra used for the estimation of the model parameters.

Power spectra have been computed by using the Welch's average modified periodogram method [24]. In particular, the model Power Spectral Density (PSD) was computed using simulated signal with duration 100 seconds, and averaging 50% overlapping sections each with duration 1 second. The use of a 100 seconds simulated signal is justified by the necessity to reduce the variance of the estimated spectrum to an acceptable level. We verified, using a random repetition of the same simulation by changing the input noise, that these spectra are only scarcely affected by the single noise realization. All power spectra have been preliminary normalized to have unitary area in the same frequency range (6–50 Hz). Since the signal beyond 40 Hz may be corrupted, the limit of our investigated gamma range was 30–40 Hz. In particular, we did not investigate the so-called high-gamma range (above 50 Hz).

We examined 5 subjects with spinal cord injury (SCI; 4 males, 1 female, mean age 26.4 ± 2.8 years) and 5 healthy subjects (4 males, 1 female, mean age 25.1 ± 1.5 years). Informed consent was obtained from all the subjects. The study was approved by the local ethics committee. The SCIs were all of traumatic aetiology and were located at the cervical level (C6 in 3 SCI subjects; C5 and C7 in the remaining 2 subjects); at the time of the study, all the patients had a stabilized lesion (mean time since trauma 19.4 ± 7.2 months). Neurological status was assessed according to the American Spinal Injury Association (ASIA) standards on the basis of the patients' motor and sensory scores, neurological level, and neurological impairment. The completeness of the lesion was defined according to the concept of sacral sparing: sensory preservation of the peri-anal zone and/or motor function of the external anal sphincter (preservation of the lower sacral segments). The lesion was complete in all 5 patients (ASIA-A: complete motor and sensory loss below the lesion level). None of the SCI patients had suffered a head or brain lesion in concomitance with the spinal injury. Neither uncontrollable spasticity-induced body movements nor dysaesthetic pain syndrome were reported by any of the patients. All subjects were right-handed as assessed by the Edinburgh inventory [25].

In order to perform a subsequent fitting, we chose only those EEG tracings for which alpha and gamma rhythms were located at approximately the same frequencies in the three ROIs. This corresponds to model hypothesis (see in what follows) that each of these rhythms is generated by a single external source (limitations of this choice will be discussed at the end). 102 tracings satisfied this criterion. The algorithm was able to fit 59 of these trials: 36 trials on healthy subjects and 23 trials on tetraplegic ones.

### 2.4. The Model of the Motor Task

Analysis of real EEGs (see also [20, 21]) demonstrates that power spectral density during the task may exhibit three simultaneous rhythms, in the alpha, beta, and gamma ranges, respectively. In order to simulate this behavior, we assumed that the cortical ROIs involved in the movement (i.e., the M1F_L, the CMA_L, and the SMAp_L), when activated, oscillate with an intrinsic rhythm in the beta range. This hypothesis reflects the frequent idea that, during behavioral activation, beta rhythms are generated locally, perhaps by a recurrent feedback loop involving pyramidal cells and inhibitory interneurons [26]. These waves represent excitement of the cortex to a higher state of alertness or tension. Moreover, we assumed that the alpha rhythm is sent to the cortex by an external area (probably located in the thalamus and reticular nucleus). This hypothesis corresponds to the idea [32, page 201] that alpha rhythms arise from the endogenous rhythmicity of thalamic populations, which are then transmitted to other thalamocortical populations even in the absence of an external stimulus. Finally, an important problem is how to produce gamma rhythms in the model. A first possibility is that all ROIs can generate not only their intrinsic beta rhythm, but also a gamma oscillation, via a second group of populations with faster kinetics, and that these gamma rhythms are then synchronized via long-range synapses. The idea of multiple rhythms in the same ROI was proposed by David and Friston [15], and was used by us in a previous model for connectivity estimation [17]. A second possibility, which allows PSD to be mimicked with a smaller number of parameters, is that gamma oscillation is generated by a single far region of the cortex, and then transmitted to the other ROIs via long-range synapses.

**Figure 1:**
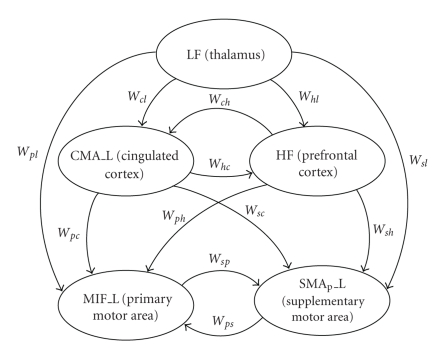
Model of interconnected ROIs used in the present work to simulate power spectral densities in prefrontal regions during a foot-movement task. *W*_*ij*_ are connectivity weights, estimated from real data using the genetic algorithm described in the text. The regions CMA_L, M1F_L and SMA_p__L oscillate in the beta range when stimulated with white noise. The LF region oscillates in the alpha range, whereas the HF region generates a rhythm in the gamma band (see Table 1  for parameter numerical values within the regions).

In the present study we adopted the second hypothesis. First, we assumed that the thalamus receives an external input (simulated as a significant white noise term) and drives the other populations but does not receive any connectivity from them (i.e., any possible feedback from the cortex to the thalamus is neglected). Hence, the motor command originates from the low-frequency region (LF), and spreads toward the cortex. Moreover, the three ROIs in the cortex (CMA_L, M1F_L, and SMAp_L) can recruit a gamma or high-frequency rhythm from another region (named HF), which may be located in the prefrontal cortex. This rhythm should reflect the cognitive or conscious aspects of the task. Finally, the cingulate cortex can also modulate the HF region and drives the other two ROIs (i.e., the primary and supplementary motor areas). The latter are linked via a feedback loop. A sketch of the overall model is illustrated in Figure 1.

### 2.5. The Model Parameters

The model has a relatively large number of parameters, but only a few of them were used as variables for the fitting procedure. It appears that letting the fitting algorithm modulate all of the model parameters leads to incoherent solutions: the same simulated power spectra can be obtained with different sets of parameters. So the parameters estimated by the fitting algorithm were only the reciprocal of time constants of excitatory synapses (to tune power peaks frequencies) and connectivity strengths (to adjust power peaks relative amplitudes). The other parameters have constant values, given in Table 1. Most of these values are biologically plausible [11] and let the model oscillate in the alpha (8–12 Hz), beta (12–30 Hz), and gamma band (>30 Hz) [18, 19]. Still the input mean *m* and variance *σ* ^2^ have been estimated via the fitting procedure, since no plausible values for these parameters have been found yet. In fact, as usual in neural mass models [12, 15, 20], this noise simulates all random contributions coming from external sources not included in the model and also accounts for internal neural variability. To do this we run a preliminary set of fittings in which *m* and *σ* ^2^ were used as fitting variables in order to find their optimum values for each trial. The values found were averaged and used as constants (Table 1) in the following fitting procedures.

### 2.6. Genetic Algorithm and Fitting Procedure

A Genetic Algorithm (GA) is a search technique that solves optimization problems by simulating the Darwinian natural selection [27]. We used the GA to find the set of model parameters for which the model output fits a given real EEG signal. Parameters used for the fitting procedures are the reciprocal of time constants of excitatory synapses (*a*_1_), and connectivity strengths.

**Figure 2:**
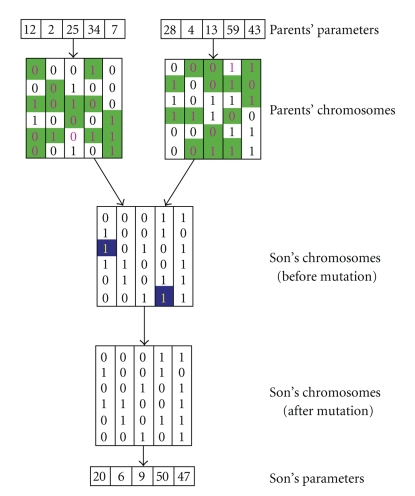
An exemplum of the mechanism for son generation implemented in the genetic algorithm.

The GA is divided into generations. Each generation consists of a lot of individuals that are candidate solutions (sets of model parameters) for the fitting. The first generation is typically random. Parameters are represented as bit arrays (chromosomes). Each individual is ranked with a fitting coefficient (FC) in the range [0, 1] by calculating the model output and comparing it to the real EEG signal: the better the fitting between the simulated signal and the real one, the higher the FC of the individual. Best-ranked individuals (higher FC) have higher probability to reproduce. During reproduction couples of parents are randomly selected according to their FCs. Each couple generates two new individuals whose chromosomes are obtained from applying genetic operators to the parents' ones. Typical genetic operators are crossover and mutation (Figure 2). Crossover is the exchange of genetic material between parents to generate the sons' chromosomes. Mutation simply switches the values of a low percentage of bits (mutation rate). The worst individuals of the previous generation are replaced with the best newborn individuals. In this way, each generation tends to preserve the best genetic material. The algorithm converges to a population composed of sets of parameters that fit the real EEG signal well.

The major challenge in implementing a GA is to find an efficient fitting function for determining the FCs and rank the individuals, so that the algorithm is able to converge in reasonable time. To compare the simulated signal to the real one, we used their PSD. Actually, analysis of the peak frequencies and amplitudes in the PSD allows evaluations of the rhythms characterizing the signal, their frequencies, and the relative power associated to each frequency band.

We introduced some changes to the original GA to improve its speed of convergence.

- The global population was divided in 4 tribes. Each tribe has its own fitting function. The algorithm allows migration between tribes, so that each individual may choose the tribe that consent its offspring to converge to the solution in the fastest way. In order to compute the FCs of each tribe, we first calculated three alternative cost functions. The first is the classic mean square error. The second aims at quantifying the similarity in the ratios between the local maxima and the local minima (i.e., it gives more emphasis to the maxima and minima of the PSD than to other values of the PSD). The third focuses the attention especially to the position of the peaks (i.e., the frequencies of the three rhythms). These three functions were then combined with different weights, in order to obtain four alternative FCs to be used in the four tribes. The fourth tribe (also named *melting pot*) is the one characterized by the strictest requirements.
- The algorithm uses a dynamic mutation rate. The probability of a bit to switch is related to the similarity between the parents' chromosomes: the more similar the chromosomes, the higher the mutation rate. A high similarity between parents means that the population converged to a local maximum; in this condition, an increase in the mutation rate would favour the escape from the maximum attraction field.
- An aging factor was introduced. This means that members of the previous generation can still generate sons and daughters, but starting with a decreased FC. Otherwise, the creation of new populations would erase all good old individuals, and if they had poor sons and daughters their legacy would be lost. On the other hand, if old individuals are not weakened, evolution may be too slow.
- The order of bits inside chromosomes can be shuffled. Commonly each parameter is encoded in a single chromosome, but such a coding system is inefficient when combined with the dynamic mutation rate described above. When one of the parameters approaches its best value, it tends to be inherited by all the members of the population. This means that all the individuals have an almost identical chromosome, thus the mutation rate for the bits encoding this parameter grows rapidly and the partial information reached may be wasted in the successive generation. This problem can be avoided by spreading the information of each parameter among all the chromosomes. Figure 2  illustrates a more standard coding system.

The algorithm stops either when individuals finish improving their FCs, or after 400 generations. At the end of the simulation the best individual belonging to the melting pot is taken as the best solution.

We noticed that the most beneficial changes are those which best resemble the natural selection.

## 3. Results

**Figure 3:**
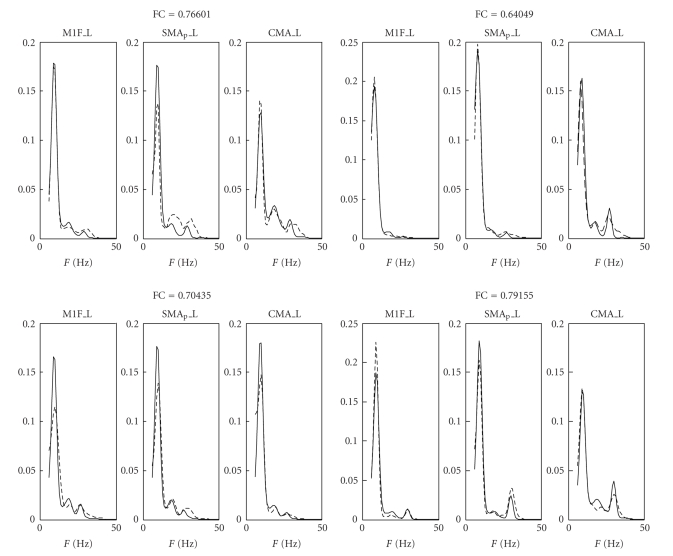
Comparison between real (dashed line) and simulated (continuous line) power spectral densities in the three regions M1F_L (primary motor cortex), SMA_P__L (supplementary motor cortex), and CMA_L (cingulate cortex) of the left hemisphere during execution of the foot imagery motor task. The left panels refer to two healthy subjects, while the right panels refer to two tetraplegic patients. All spectra are normalized to have unitary area in the range 6–50 Hz. The value of the fitting coefficient (ranging between 0 and 1) is shown above each panel.

Exempla of model fitting in four exemplary cases are shown in Figure 3. The left panels refer to two healthy subjects, while the right panels refer to two tetraplegic patients. It is worth noting that the model is able to simulate the position and the relative amplitude of the three peaks in all three ROIs quite well. The other fitted PSDs are similar to those presented here, both for what concerns the shape and the quality of fitting.

**Figure 4:**
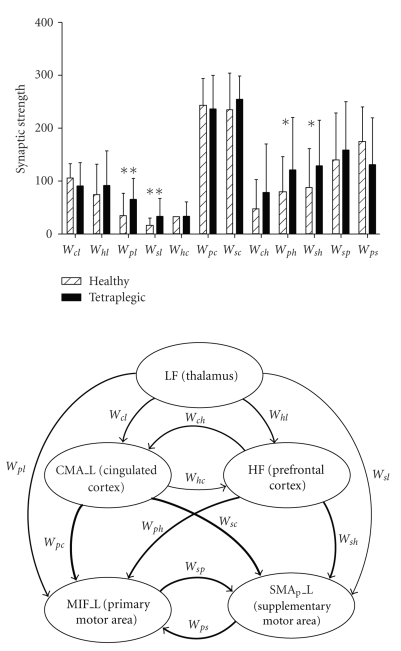
Connectivity weights (mean value + SD) estimated on 5 healthy subjects and on 5 tetraplegic patients with the genetic algorithm described in the text. A qualitative exemplum of the resulting connectivity, based on the average values on the entire population, is depicted in the bottom panel, where line thickness is proportional to the connectivity weight. It is worth noting in the upper panel the presence of very significant statistical differences (*p* < .01, columns with ∗∗) between healthy subjects and tetraplegic patients for what concerns the connections from the LF region to the primary motor and supplementary motor areas. Significant statistical differences (*p* < .05, columns with ∗) are also evident in the connections which link the HF region to the primary motor and supplementary motor areas.

The average values of estimated synaptic weights in the healthy population and in the tetraplegic patients are shown in the histogram of Figure 4. Two main aspects of this figure deserve attention.

First, by considering the overall fitting parameters, without distinguishing between healthy and tetraplegic subjects, one can observe that some weights are predominant compared with others. In particular, the stronger connections are those from the cyngulate cortex to the primary motor cortex, and from the cyngulate cortex to the supplementary motor cortex. A visual summary of the synaptic strengths, computed by using the average parameters in both populations, is shown in the bottom panel of Figure 4.

Second, from a separate parameter estimates, one can detect statistically significant differences in the synaptic strength between healthy and tetraplegic subjects. In particular, connectivity in tetraplegic patients is about 12% higher (on average) compared with that of healthy volunteers. Differences in connection weights between the two classes are very significant (*p* < .01 evaluated with an untailed *t*-test) from the thalamus to the primary motor cortex and from the thalamus to the supplementary motor cortex. The differences in the connection weights are also significant (*p* < .05) from the high frequency region to some cortical ROIs.

**Figure 5:**
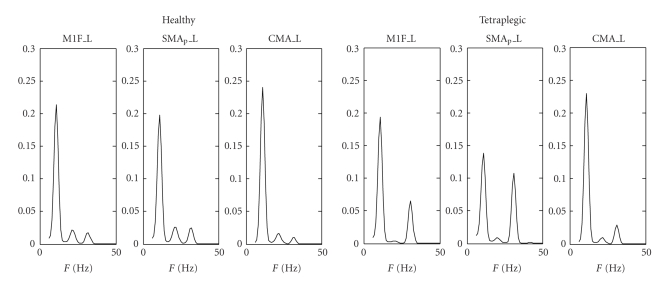
Example of paradigmatic power spectral densities simulated with the model using the average connection weights estimated on healthy volunteers (left panel) and on tetraplegic patients (right panel). All spectra are normalized to have unitary area in the range 6–50 Hz. It is worth noting the higher peak in the gamma range, and the lower peak in the beta range in tetraplegic patients compared with the healthy subjects.

Finally, we used the average values of the synaptic strengths in the two populations to compute paradigmatic PSDs (one for a typical healthy subject using the average parameters of that class and the other for a typical tetraplegic subject). The results are illustrated in Figure 5. As it is evident from this figure, the paradigmatic tetraplegic subject exhibits a stronger peak in the gamma band compared with that evident in the paradigmatic healthy volunteer and a smaller peak in the beta range. This difference is a consequence of the higher connectivity weights from the HF region and from the LF region.

## 4. Discussion

The aim of this work was to derive patterns of connectivity among the main regions of interest (the cingulate cortex and the primary and supplementary motor areas) involved in simple motor tasks. To this end, we used neural mass models and electrophysiological data obtained with scalp EEG, propagated to the cortex. Moreover, we analyzed differences between normal and tetraplegic subjects. Although various attempts to derive connectivity from EEG, and to characterize EEG in pathological conditions are present in the literature, most works make use of empirical model (e.g., based on coherence and correlation among time series). Just a few attempts to elucidate existing data via interpretative models can be found in the literature [15–17].

In an interpretative model, parameters have a clear biophysical significance, and the model allows the formulation of hypotheses on the physiological mechanisms, the neural architecture, and the parameter changes responsible for data generation. Promising models assume the presence of interacting neural masses, which are reciprocally connected and generate the neural signals responsible for the measured electrical activity. Similar models integrated with Bayesian inference (a framework named “Dynamic causal models” by the authors) were used by Friston and coauthors to estimate effective connectivity from neuroimaging data [15, 28], to analyze event-related potentials [29] or to predict the spectral profile of local field potentials in the rat [17]. Neural mass models were used to study the transition to seizures and to model epileptic activity [12, 30], to analyze the effect of drugs on EEG spectra [31], or to simulate the effect of the overall brain connectivity on individual EEG rhythms measured on the scalp [16].

Our work goes in the same direction as previous papers. However, three main innovative methodological aspects deserve a critical discussion: the kind of information used to validate the model, the structure adopted for the model, and the fitting procedure for parameter estimation.

The first important issue concerns what kind of data the model is intended to reproduce, and so, which measurement is compared to model output. This is a crucial point, since the type and structure of a model are strictly dependent on the problem under study. In this work, as in previous ones [18–21], we focused attention on the frequency content of cortical EEG, in particular on the peaks of power spectral density. Indeed, spectral measures are commonly used to summarize cortical dynamics and to assess changes in cortical activity during cognitive and/or motor tasks. It is generally believed that the alpha rhythm originates from the thalamus and is distinctive of a relaxed state. The beta rhythm is associated with normal waking activity, as it occurs during natural human motor behavior or after proprioceptive stimulation. A shift from alpha to beta rhythms is considered a marker of alerting. Gamma rhythms appear to be involved in higher mental activity, including perception and consciousness. Although these rhythms are currently described and analyzed in the neurophysiological literature [32, 33], the problem of how to link their changes to the underlying neural processes, the neural architecture and connectivity strength is still largely unsolved.

An important aspect is that we focused attention just on three ROIs, and we never tried a fitting to other ones. The ROIs were selected according to widely accepted considerations on their involvement in the preparation and execution of simple self-generated movements. In fact, there is a general consensus that the M1F and the medial aspect of the SMA_p_ are amongst the main generator sources of the early and late components of the motor-related cortical potentials (reviewed by [34]) which, in turn, reflect the physiological excitation of the cortical areas involved in preparing and producing movements. Anatomical and physiological studies on nonhuman primates have demonstrated that among the distinct cingulate motor areas buried in the cingulate sulcus, those roughly located at the same rostrocaudal level as the SMA_p_ proper (caudal CMA, dorsal, and ventral parts) are primarily implicated in movement execution itself rather than in higher cognitive control of voluntary movements (for review see [35, 36]).

In order to simulate EEG spectral patterns in these areas, including both alpha and beta as well as gamma rhythms, we adopted a simple model structure based on a few a priori assumptions. First we assumed that the cingulate cortex drives the primary motor area and the supplementary motor area during execution of the task, but it receives only negligible feedback from them. This assumption seemed justified by the attention that the cingulate cortex has received in the neuroscientific literature recently [37]. In these contributions the cingulate cortex is seen as a part of the cortex, that is, mainly involved in the promotion of action and movements of decisions. By contrast, the two motor areas may be connected by a reciprocal feedback. These areas are important in our model since the primary motor area is responsible for the execution of all voluntary movements, while the supplementary motor area implements internally generated or well-learned actions, that is, actions which do not require monitoring the external environment.

A further assumption is that the three ROIs under analysis, if stimulated, can oscillate with an intrinsic beta rhythm. This assumption agrees with present knowledge. Indeed, as traditionally described in the literature, a motor related activity in the beta range is frequently located close to the sensory motor area following finger movements [38] and is reflected to the premotor area [39]. As suggested by [40] beta oscillations may be “indicative of a resonant behavior of the connected networks in the sensorimotor areas.” This reflects our basic model assumption.

Beyond this fundamental aspect, the model incorporates two other important assumptions, which are used to generate alpha and gamma rhythms, but have a less evident physiological and neural counterpart.

First, model assumes that a low frequency alpha rhythm originates from an external area (that we named “thalamic area”) and then propagates to the other regions of interest. Indeed, a classic idea on the genesis of alpha rhythms [32, page 201] is that this rhythm arises from the endogenous activity of thalamic neurons, or from thalamocortical connections, especially involving the occipital region. Recent works on the cat, support the critical role of the thalamus for the generation of occipital oscillations [41]. A recent study on the location of EEG rhythms in humans confirms that alpha rhythms are especially evident in the occipital or occipito-temporal regions, that is, they mainly arise from posterior neural sources [39]. Hence, although we cannot exclude that a source of alpha rhythms may also be present in the examined frontoparietal regions, the most likely hypothesis is that this rhythm originates in thalamic and/or occipital regions, and is then transmitted toward the other regions of interest.

An important simplification, which deserves a brief comment, is that we neglected any feedback synapse from cortical regions to the “thalamic area.” Of course, cortico-thalamic feedbacks exist in the brain and may have a role in the modulation of the alpha spectral content. Our choice has been adopted just to reduce the number of parameters in the fitting procedure, in order to avoid the problem of “overfitting.” In fact, increasing the number of unknown parameters improves the quality of fitting, but worsens the reliability of parameter estimates.

A further important assumption is that also the gamma rhythm originates from an external area, that we supposed to be located in the frontal cortex. This hypothesis is corroborated by the observation that neurons in the frontal cortex shows the intrinsic capacity to oscillate at 40 Hz [42, 43]. However, alternative hypotheses on the origin of gamma rhythms can be found in the literature (see [44] for an excellent review) and we cannot exclude that this rhythm originates internally in the considered ROIs due to recurrent excitation and inhibition mechanisms (especially involving fast inhibitory interneurons). Hence, the gamma region in the model should be considered as a “latent source,” that has not necessarily a physiological counterpart. This problem requires further theoretical and experimental work.

Once this model structure has been designed, a fundamental point concerns what aspects of the spectra should be used to perform a best fitting between model predictions and real data. In previous works we used a least-square criterion function of the difference between model and measured spectra [18–21]. Assuming a Gaussian distribution of the measurement error, a least square criterion corresponds to a maximal likelihood estimation, that is, maximization of the a priori conditioned probability. A more complex Bayesian procedure has been adopted by Moran et al., recently [17] under the framework of dynamic causal models [28, 45]. A Bayesian procedure involves also the inclusion of some a priori knowledge on the probability distribution of the estimated parameters.

In the present work we tried an innovative strategy, based on the idea that not all aspects of the PSD are of equal interest. In particular, we focused attention especially on the position and relative amplitude of the main peaks in the power spectra, thinking that these summarize the underlying mechanisms generating EEG rhythms. Moreover we tried different complementary “cost functions” in the implementation of the genetic algorithm (GA). Although GA are time consuming compared with other minimization techniques, they offer the possibility to try different alternative solutions for the problem (implementing different tribes) and to overcome the problem of local minima (which often makes the result of fitting procedures untenable) by generating different sons through mutations in the parameter space.

Two main objectives have been pursued with this technique: to discover possible simple circuits, connecting the three aforementioned ROIs, able to explain the observed PSDs, and to detect possible differences in connectivity circuits between healthy subjects and tetraplegic patients. Results point out the existence of significant differences between the two classes, especially for what concerns the weights which link the LF (thalamic) and HF regions to the primary and the supplementary motor cortices. In particular, these weights are stronger in tetraplegic patients compared with healthy individuals and these differences are statistically significant. Differences in connectivity weights might reflect a higher awareness (related with the gamma component) and a greater attention (related with thalamic inputs) in the tetraplegic patient than in the normal individuals, that is, greater concentration toward the task. The existence of larger and stronger connectivity weights in the cortical connectivity networks estimated in tetraplegic patients compared with those estimated in healthy volunteers has been previously observed by several authors [46, 47].

A further interesting result of our work is that the greatest weights in the neural circuit are those which link the cingulate cortex to motor areas. This result underlines the importance of a feedforward signals from the frontal cortex in the initiation and planning of the voluntary movement.

In the present work we performed 12 statistical tests, hence a possible objection is that the significance level should be corrected to account for multiple hypotheses. The problem of whether correction is appropriate or not is quite complex and depends on the objective of the work. As clearly stated in recent publications [48] if the main goal is generation hypothesis or initial screening for potential solutions, it may be appropriate to use the standard significance level without corrections to avoid Type II errors (not detecting real differences or trends). Conversely, if the main goal is rigorous testing of a hypothesis, then an adjustment for multiple tests (like Bonferroni or Holm's methods) is needed. The objective of the present work is certainly “hypothesis generation,” hence we preferred to use classical *t* test to avoid type II error. Of course, in order to “test the hypotheses” generated with our procedure, one needs to repeat the experiment with new “fresh” data, considering only the individual hypotheses to be verified, and using a correction. This may be the subject of future works.

Finally, it is important to discuss the main limitations of the present preliminary work, and possible lines for future changes.

A first aspect concerns the variability of parameter estimates within the same subject. Although this variability is less accentuate compared with that between the two classes, and between different subjects in the same class, it is still quite elevated. Analysis of how the connectivity pattern may vary in the same subject from one trial to the next still requires a deeper future analysis.

In the present model we assumed that connectivity originates from pyramidal neurons, and reaches the input of excitatory interneurons, that is, we did not consider possible lateral connections from pyramidal neurons to inhibitory interneurons. Inhibitory interarea connections, however, may be important to reduce neural activity, to avoid instability, and to improve synchronization among rhythms. Lateral inhibitory synapses were considered by David et al. [29], and Stephan et al. [28], in their DCM schema of neural populations for the analysis of event related responses. In particular, these authors assumed that lateral connections originating from pyramidal neurons target to all other populations (both excitatory and inhibitory) in the lateral ROIs, although they did not consider the presence of inhibitory interneurons with fast kinetics. Inclusion of lateral connections toward inhibitory interneurons may be of value in future works, to improve two aspects of results. First, it may help to maintain the activity of the motor and premotor ROIs far from saturation. Indeed, with the present values of parameters, these two populations are strongly activated and often work close to the upper saturation region of their sigmoid. Second, activation of fast inhibitory interneurons might help to explain the presence of gamma rhythms, even without introducing an ad hoc rhythm from an external population. The idea that gamma rhythms may originate from stimulation of fast inhibitory interneurons (or alternatively from gap junctions) has been proposed by various authors recently [32, 49]. Of course, a flaw of introducing lateral synapses to inhibitory interneurons is the increase in the number of free parameters, which may further complicate the convergence of the fitting procedure and the interpretation of results.

Another important limitation of the present work is that the model is able to simulate PSD spectra only if the rhythms in the three ROIs (in the alpha and gamma bands) have almost the same frequency. In view of that, we excluded all trials which present different frequencies in the spectra from the best fitting procedure. The reason for this limitation is that the three ROIs receive the alpha and gamma oscillations from the same external ROIs (i.e., the alpha rhythm from the LF region or thalamus; the gamma rhythms from the HF ROI, prefrontal, see Figure 1). In order to generate rhythms with different frequencies in the alpha and gamma bands, one should hypothesize the presence of more LF and HF regions. However, this aspect would further complicate parameter estimation and would make the model less parsimonious. It is possible that introduction of lateral interregion synapses directed to inhibitory interneurons may allow a more flexible positioning of rhythms in individual ROIs.

Finally, we are aware that use of the genetic algorithm, although very flexible in finding a good solution avoiding local minima, is time consuming. Alternative more efficient fitting methods (maybe introducing some prior probability for the estimate, according to a Bayesian approach [50]) may be attempted in future studies.

In conclusion, the present work represents a first attempt to explain the presence of multiple rhythms in three ROIs involved in motor tasks, and their variability, using a simple model of interconnected populations. Encouraging results concern the capacity to obtain reliable PSD spectra, by acting on a few parameters representing the connection weights, and to detect significant differences between the two classes. However, important limitations are still evident: they are especially concerned with a lack of inhibitory interactions among ROIs, with the dispersion of individual parameter estimates, and with the difficulty to generate more flexible peaks in the spectra. Overcoming these limitations deserve much future work.

Nevertheless, despite their present limitations, we claim models of interacting neural mass may be of great value to gain a deeper insight into the mechanisms of rhythms generation in EEG, and to start the formulation of more quantitative hypotheses on the neural architecture and connectivity changes underlying motor/cognitive tasks.

## Figures and Tables

**Table 1:** Model parameters.

Parameters	LF	CMA_L, M1F_L, SMAp_L	HF
*A*(*mV*)	2.67	5.17	5.55
*B*(*mV*)	3.15	4.45	3.8
*G*(*mV*)	22.3	57.1	173
*b*_1_(*s* ^−1^)	20	30	40
*g*_1_(*s* ^−1^)	300	350	790
*m*(*mV*)	−103.3011	−130.4829	−16.1439
*σ* ^2^(*mV* ^2^)	27807	10028	23642

		All Regions	

*C*		135	
*C*_1_		*C*	
*C*_2_		0.8* C*	
*C*_3_		0.25* C*	
*C*_4_		0.25* C*	
*C*_5_		0.3* C*	
*C*_6_		0.1* C*	
*C*_7_		0.8* C*	
*r*(*mV* ^−1^)		0.56	
*s*_0_(*mV*)		6	
*e*_0_(*s* ^−1^)		2.5	
